# Integrating Physical and Topographic Information Into a Fuzzy Scheme to Map Flooded Area by SAR

**DOI:** 10.3390/s8074151

**Published:** 2008-07-10

**Authors:** Nazzareno Pierdicca, Marco Chini, Luca Pulvirenti, Flavia Macina

**Affiliations:** Department of Electronic Engineering, Sapienza University of Rome, via Eudossiana 18, 00184 Roma, Italy; E-Mails: nazzareno.pierdicca@uniroma1.it (N. P.); pulvirenti@die.uniroma1.it (L. P.)

**Keywords:** SAR, Flood, Data fusion, Fuzzy

## Abstract

A flood mapping procedure based on a fuzzy sets theory has been developed. The method is based on the integration of Synthetic Aperture Radar (SAR) measurements with additional data on the inundated area, such as a land cover map and a digital elevation model (DEM). The information on land cover has allowed us to account for both specular reflection, typical of open water, and double bounce backscattering, typical of forested and urban areas. DEM has been exploited to include simple hydraulic considerations on the dependence of inundation probability on surface characteristics. Contextual information has been taken into account too. The proposed algorithm has been tested on a flood occurred in Italy on November 1994. A pair of ERS-1 images, collected before and after (three days later) the flood, has been used. The results have been compared with the data provided by a ground survey carried out when the flood reached its maximum extension. Despite the temporal mismatch between the survey and the post-inundation SAR image, the comparison has yielded encouraging results, with the 87% of the pixels correctly classified as inundated.

## 1. Introduction

Mapping the extension of an inundation is fundamental for relief organization and to assess the damages. Remote sensing data are useful tools in this field, especially for events occurring in remote regions or in areas characterized by lack of rain-gauge stations, where hydrological information are difficult to be gathered. Among remote sensing sensors, Synthetic Aperture Radar (SAR) offers the advantage of getting high spatial resolution images in almost all-weather conditions, as opposed to passive instruments operating at infrared and visible bands. This feature is particularly attractive and in most cases essential, since flooded areas are often obscured by heavy cloud coverage. The change detection potential of SAR, based on variations of the backscattering coefficient *σ^0^* (i.e., the image intensity), or decrease of coherence derived from SAR image pairs (i.e., the interferometric phase), can be successfully exploited to remotely map inundations [1-3]. For instance, the Mississippi flood of 1993 [4], the 1996 and 1997 inundations in the Red River Valley [5-7], the Elbe event of mid-August 2002 in Germany [8], the Oder River inundation of 1997 [9] and the Yangtze River flooding occurred in China in summer 1998 [1] were monitored by using SAR images.

The effect of an inundation may produce different changes in the SAR image, depending on the type of underlying terrain. Flooded bare soils have lower backscattering with respect to the surrounding non-inundated areas, since a smooth water surface acts like a specular reflector [2], and the flood detection is generally carried out by applying thresholds on a SAR image recorded after the event [10]. Note that wind roughening increases the backscattering from the inundated surface, thus lowering the contrast between flooded and non-flooded areas. On the contrary, inundated forest areas generally produce a large radar return, caused by a double bounce backscattering mechanism between the water surface and the trunks [11-12]. In this case, the difference between two SAR images, recorded before and after the event, is analyzed to identify areas where an increase of *σ^0^* occurs. The double bounce mechanism may produce a significant radar return in inundated urban areas too.

Methods based on thresholds applied to a SAR image were widely adopted in past investigations. Henry et al. [8] determined the thresholds by analyzing the histograms of both Envisat ASAR and ERS-2 observations of the Elbe river flood occurred in 2002. Cunjian et al. [13] applied a threshold to a RADARSAT image concerning an event occurred in China in 1998, and used a digital elevation model (DEM) to distinguish the dark shadow due to relief from water. Good results were achieved by using a SAR polarimetric system. For instance, a decision tree based on thresholds applied to multipolarization L-band and C-band SIR-C data concerning Amazonia was developed by Hess et al. [14] to discriminate five types of land cover, included water and flooded forest.

Algorithms founded on sophisticated segmentation techniques were also developed to delineate the boundaries of a flooded area. An example is the active contour model, adopted by Horritt et al. [2]. In their study, the segmentation was applied to an ERS-1 image and to a coherence map built by employing two ERS-1 images collected before and after the flood of the river Thames in 1992. Better results were obtained by using other data, such as a digital terrain model (DTM) derived from airborne laser altimeters (Lidar), together with SAR data [15]. For the events of the River Alzette floodplain on 2003 and the River Mosel on 1997, SAR flood extent and a high-resolution floodplain DEM were joined to compute flood depths [16].

Our concern is that the shortcoming of most procedures is represented by the simplistic image processing methods that are used, generally based on fixed thresholds, which do not account for complications in SAR imagery due to the presence of vegetation or urban areas. On the other hand, advanced image processing procedures do not incorporate any prior information on the physics of surface scattering. Horrit et al. [2] stated that an improvement of the flood maps accuracy can be expected through the adoption of a more model based approach, rather than a heuristic segmentation method.

In this paper, we propose the application of the fuzzy theory [17] for flood boundary delineation from SAR images. The fuzzy sets basically represent an extension of the classical notion of set. While in classical set theory an element either belongs or does not belong to the set, elements of a fuzzy set have degrees of membership. These degrees are described by a membership function whose values are real numbers in the interval [0, 1]. The fuzzy theory is suitable for representing the sets for which the definition of a membership criterion is a difficult task. This is the case of the set of flooded pixels in SAR images. Even though the fuzzy logic has been widely used in the past for image elaboration and segmentation (e.g., [18-19]), to our knowledge this is the first attempt to apply such a method for flood mapping.

Since the imaging of the water surface is complicated by factors such as wind roughening and the presence of vegetation [2], as previously discussed, several pieces of information should be included in the classification algorithm to improve the reliability of inundated area maps. The use of a fuzzy-based method has also allowed us to integrate in the classification procedure prior information (e.g., DEM of the involved area, land cover map), simple hydraulic considerations which are generally neglected in flood mapping methods (e.g. the dependence of inundation probability on surface characteristics), and contextual information.

We have considered a case study concerning a flood occurred in the Alessandria district (Northern Italy) on November 5th-6th 1994. A pair of ERS images collected before (October 3rd) and after (November 9th) the inundation has been used, together with a DEM of the area and a land cover map (CORINE land cover). The result of our procedure for flood boundary delineation has been compared to a ground survey that refers to the maximum extension of the flood, whilst the post-flood SAR image has been collected few days later. Despite of this difficulty, the flood map derived using the proposed procedure seems to be able to identify the presence of water, better than a simple thresholding procedure.

In Section 2 a preliminary analysis of the available data is described, whereas Section 3 depicts the adopted fuzzy approach. In Section 4, the results of our procedure are discussed and Section 5 draws the main conclusions.

## 2. Dataset analysis

In November 1994, because of heavy rainfall, the Tanaro River flooded the town of Alessandria, in Northern Italy, and the neighboring areas. The event caused a great deal of damage, with a loss of 70 lives [20]. The peak of the event occurred on November 5th-6th. Such a disaster has been studied in the past by Boni et al. [21] through a multisensor analysis particularly focused on rain rate retrieved from passive microwave radiometric measurements. An attempt to map the extension of the flood by ERS has been carried out in [20] by means of a maximum likelihood classifier, developed for non-forested areas only.

**Figure 1.**
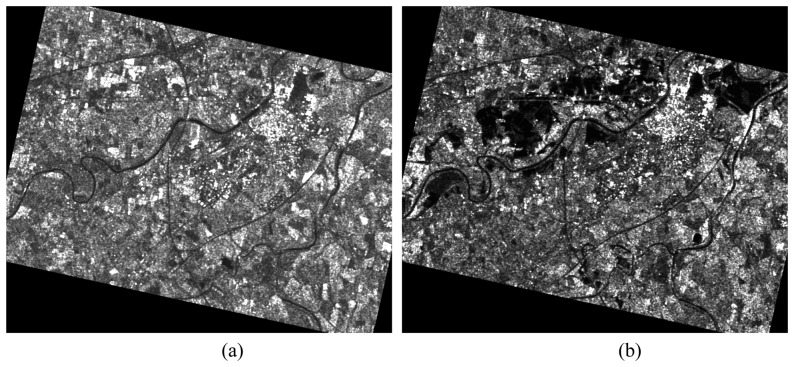
Pre (left panel) and post-flood (right panel) SAR images of the Alessandria area.

We have used a pair of ERS-1 intensity images collected before (October 3rd) and after (November 9th) the inundation. The two images, whose resolution is 12.5 m both in ground range and in azimuth, have been recorded in descending orbits. The original five look Precision Images (PRI) provided by ESA have been filtered to reduce the speckle by using a Frost adaptive filter [22], and georeferenced. They are shown in Figure 1 (pre-inundation: left panel; post-inundation: right panel). In the post-event SAR image (November 9th), some dark areas can be clearly observed. They indicate the presence of water surfaces even three days after the peak of the inundation.

**Figure 2.**
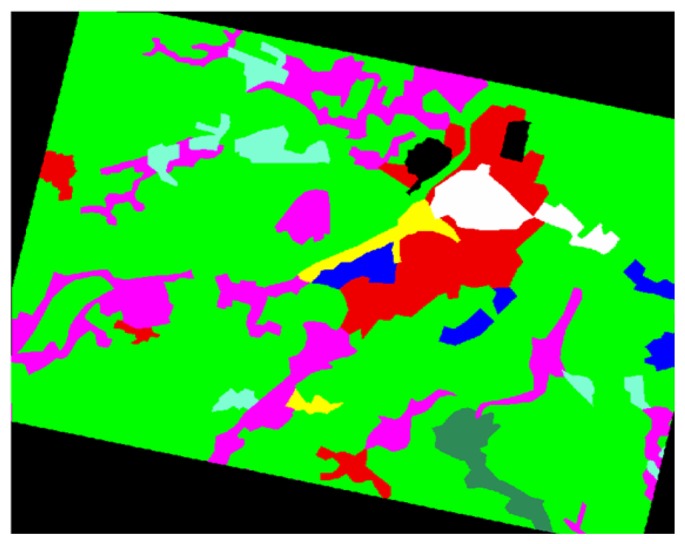
Land cover map of the area derived by CORINE database. Eight main classes are distinguished. Industrial and commercial areas (blue); cultivations (magenta); grassland (aquamarine); road and rail networks (yellow); sowable lands (green); continuous urban (white); discontinuous urban (red); woodland/shrub (sea green). Black areas are unclassified.

The orography of the Alessandria district has been characterized by a DEM with a resolution of 40×40 m furnished by Piedmont region (to which the Alessandria district belongs). The DEM has been resampled at 12.5 m pixel-spacing through a bi-linear interpolation to be co-registered with the SAR images. As for land use and land cover, we have considered the data available through the CORINE database. These data have been derived from Landsat Thematic Mapper images acquired in 1991-1992 and are shown in Figure 2. It can be observed that most of the territory (about 71%) is covered by sowable land (green). This implies the prevalence of (forward) specular reflection from the water surface, thus explaining the presence of large dark areas in the post-inundation image (Figure 1b).

The dataset has been integrated by a ground truth, provided by the local authorities, indicating the maximum extension of the flood. Unfortunately, it cannot be directly compared with the post-inundation SAR image which has been collected three days later. However, it has yielded some useful indications for validating the results.

**Figure 3.**
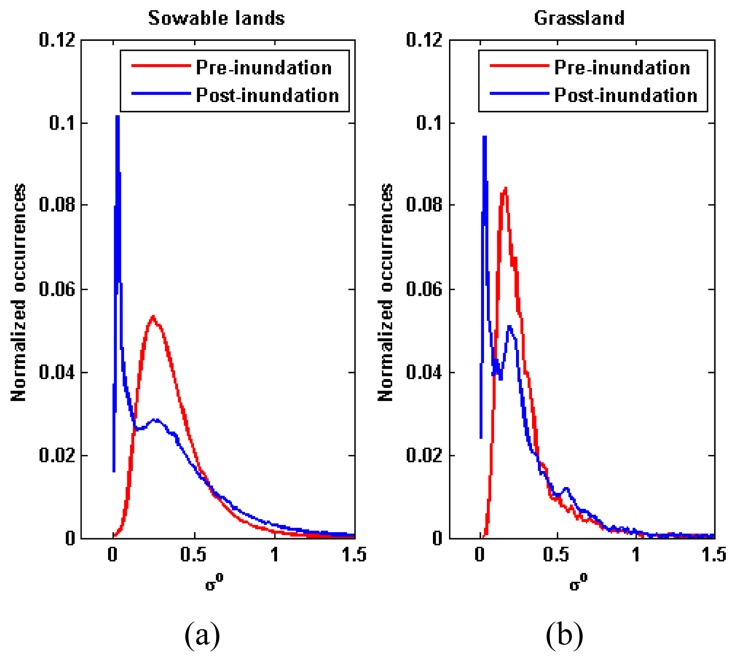
Histograms of *σ^0^* before and after the inundation. Left panel: sowable lands; right panel: grassland. Red and blue lines concern pre-flood and post-flooded images, respectively.

To study the scattering behavior of different cover classes when the land is inundated, we have performed a preliminary analysis of the dataset. We have derived the histogram and the mean value of *σ^0^* for each different type of land cover, both for flooded and non-flooded areas, distinguished according to the ground truth. Figure 3 shows the histograms for the classes of sowable lands (left panel) and grassland (right panel). The mode of the histograms related to the post-inundation image (blue solid lines) is almost at the same value of *σ^0^* (0.02 m^2^/m^2^). The same occurs for the class of cultivations (not shown). This means that, where water surfaces act as specular reflectors, the preexistent land cover does not influence the value of *σ^0^*.

**Figure 4.**
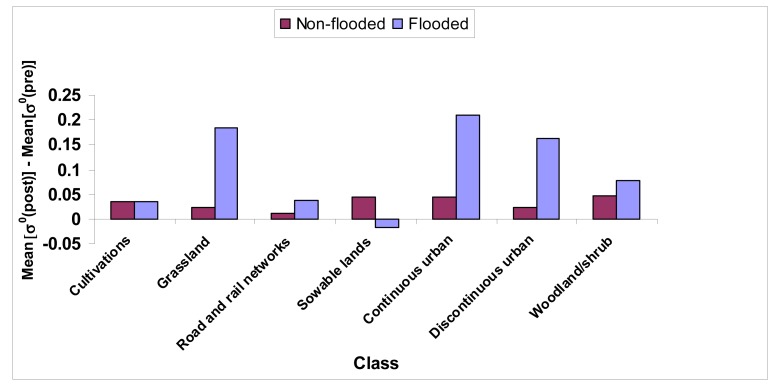
Difference between the mean values of *σ^0^* measured after and before the flood in the area near the town of Alessandria. Note that industrial areas are neglected since they were not involved in the flood, according to the ground truth.

Figure 4 shows the difference between the mean values of *σ^0^* extracted from pre-flood [*σ^0^*(*pre*)] and post-flood [*σ^0^*(*post*)] images. The classes of urban areas (continuous and discontinuous urban), rail and road networks, grassland and transitional woodland/shrub present an increase in mean[*σ^0^*(*post*)]-mean[*σ^0^*(*pre*)]. While this increase could be expected for woodland and urban areas because of the enhanced double bounce effect, it is surprising for grassland. After a deeper analysis, we have found the presence in both SAR images of some very bright pixels in the areas labeled as grassland (outside the range of abscissas in Figure 3). This may imply that manmade structures were located in this area at the time of SAR overpasses and the increase of *σ^0^* could be ascribed to double bounce backscattering. It is worth reminding that, whilst the SAR observations regard 1994, the CORINE database has been derived from data collected in 1991-1992, so that such a temporal mismatch may have caused the missing of new urban settlements in the land cover map. Moreover, it must be considered that part of the areas labeled as inundated by the ground survey were probably non-flooded at the time of the second SAR observation. Therefore, we guess that the increase shown in Figure 4 is underestimated.

## 3. Methodology

The procedure for flood boundary delineation has been designed to account for physics of the scattering mechanisms, hydraulic considerations and prior information on land cover and topography. The requirement to avoid noisy maps with isolated points has been pursued as well, by introducing the contextual information. The fuzzy approach has revealed a very valuable tool to integrate these different pieces of information.

### 3.1. The fuzzy sets

**Figure 5.**
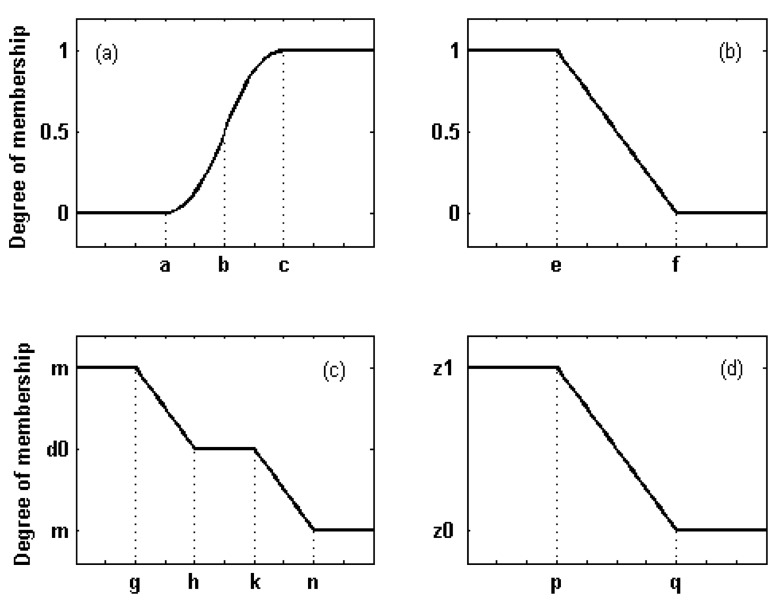
Membership functions used in this work.

We have followed a method based on standard membership functions. Pal et al. [18] proposed a function for pixel intensity, named standard S function and shown in Figure 5a (upper left panel). It is characterized by three parameters *a*, *b* and *c*, with usually *b* = (*c*+*a*)/2. According to the standard S function, the higher the intensity of the pixel, the higher is its degree of membership. We have adopted the S function to assess the membership to the flooded areas having *σ^0^*(*post*)>*σ^0^*(*pre*), i.e., urban and forested areas. As for the choice of the parameters, *b* has been computed, for each land cover class exhibiting a rise of *σ^0^*, as the mean increase in inundated zones, derived from the analysis presented in Figure 4. Subsequently, for *a* and *c*, a variation equal to 25% with respect to *b* has been supposed.

To define the membership of a pixel to the set of open water surfaces, i.e., flooded areas having low *σ^0^*(*post*), we have selected the function Z=1-S (standard Z function). In this case, the parameter *a* has been chosen in correspondence to the mode of the histograms related to the post-inundation image, which have revealed independent on land cover (Figure 3, blue lines), whilst *c* corresponds to the intersection between the histograms of pre- and post-inundation images for sowable lands (Figure 3).

**Figure 6.**
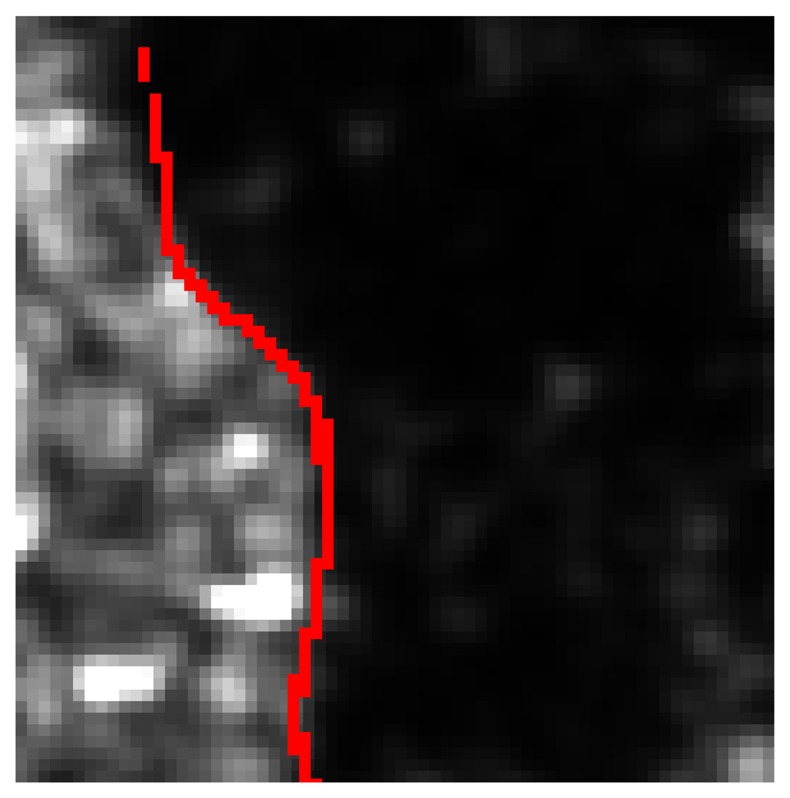
Portion of the post-inundation SAR image. A DEM contour line is superimposed (in red).

We have accounted for the information provided by the DEM too. Figure 6 shows a portion of the post-inundation image on which a DEM contour line is superimposed (in red). It can be clearly observed that the boundary of the dark area (flooded zone) exactly follows the red line, as it could be expected from very simple hydraulic considerations. A high probability of flood occurrence in low-altitude areas has been therefore supposed and the fuzzy set of the low-altitude pixels has been introduced. We have also considered that the probability of finding inundated surfaces is large in low-slope areas and in concave areas. DEM has been processed to compute slope and concavity, the latter being derived from DEM Laplacian. Correspondingly, other two fuzzy sets have been defined. The shape of the functions representing the degree of membership of the fuzzy sets introduced above is the same and is shown in Figure 5b (upper right panel). It is a piecewise linear function characterized by two parameters (*e* and *f*). For the fuzzy set of low-altitude pixels, *e* and *f* have been set equal to the minimum (80 m) and maximum (110 m) values of the heights in the area observed by SAR, whilst for the slopes we have chosen 0° and 10.3° and for the Laplacian −0.01 and 0.01.

### 3.2. The fuzzy-based method

**Figure 7.**
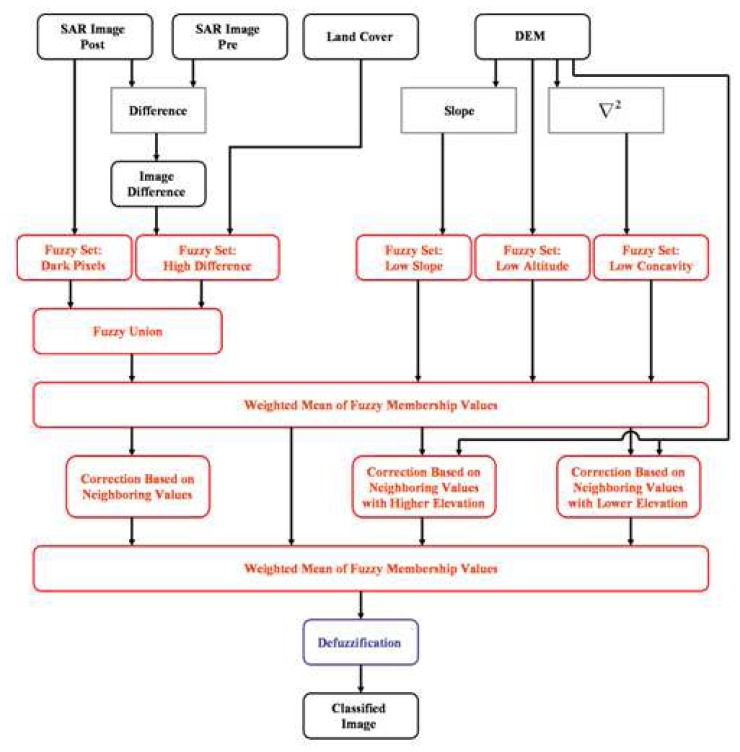
Block diagram of the fuzzy algorithm.

The block diagram of our fuzzy-based procedure is shown in Figure 7. From the fuzzy sets of the pixels with low *σ^0^*(*post*) (whose membership degree, assessed by the standard Z function, is *d*1) and of the pixels with high *σ^0^*(*post*)– *σ^0^*(p*re*) (membership degree *d*2, defined by the standard S function), a new set has been derived by means of the fuzzy union, that is, by assigning to each element the largest degree of membership between *d*1 and *d*2, i.e., max(*d*1,*d*2). This new set has been combined with the three ones obtained by exploiting the DEM information (membership degrees *d*3, *d*4, *d*5) for producing the first fuzzy set of pixels classified as inundated. To do this, a weighted average of max(*d*1,*d*2), *d*3, *d*4 and *d*5 has been accomplished, giving a larger weight to the first one, i.e. that derived from the SAR measurements, with respect to those extracted from the DEM features.

The following step of our procedure aimed at taking into account some contextual information. Simply speaking, for each pixel, a correction based on the statistics of the degree of membership of the neighbors has been performed. This second part of the algorithm is founded on the following considerations: (i) the probability of the presence of one isolated flooded pixel inside an area of non-flooded ones (or vice versa) is low; (ii) the probability of the presence of a non-inundated pixel close to inundated ones located at higher altitude is low; (iii) the probability of the presence of an inundated pixel close to non-inundated ones located at lower altitude is low.

The degree of membership of the pixels has been therefore modified according to its neighbors. To account for condition (i), for each pixel we have computed the mean value *m* and the standard deviation *s* of the degrees of membership to the class of inundated pixels in a 5×5 window around the pixel itself (whose membership degree is denoted by *d0*). Then, the quantity δ=(*d0*−*m*)(1−*s*) has been calculated and, for δ, a new membership function has been defined. It is shown in Figure 5c (lower left panel) and its parameters are *g*=−0.3, *h*=−0.1, *k*=0.1, *n*=0.3. Through this function, we have assigned the window mean degree *m* to a pixel surrounded by a uniform background (this implies *s*∼0) and having *d0* considerably different from *m*. For the opposite situation (background far from being uniform, i.e., *s*∼1 that implies δ∼0), we have retained the original degree of membership *d0*.

To account for condition (ii), we have computed, within a 3×3 window, the degree of membership *dM* of the pixels located at higher altitude with respect to the central one, having degree *d0*. Then, the membership function shown in Figure 5d (lower right panel), has been applied to *d0*−*dM*, choosing *p*=−0.2, *q*=0.0, *z*0=*d0* and *z*1=*dM*. In this way the degree of a pixel surrounded by neighbors located at higher altitude with a larger degree (probably flooded) has been increased. A similar procedure has been used to consider condition (iii). Indicating by *dm* the degree of membership of the pixels located at lower altitude with respect to the central one, the function shown in Figure 5d has been applied to *d0*−*dm*, choosing *p*=0.0, *q*=0.2, *z*0=*dm* and *z*1=*d0*. In this case we have aimed at decreasing the degree of membership of a pixel surrounded by neighbors located at lower altitude with a smaller degree (probably non-flooded).

From the three fuzzy sets created to take into account contextual information and from the first fuzzy set of pixels classified as inundated, the final set of pixels classified as inundated has been produced (see Figure 7). A weighted average has been applied giving the largest weight to the membership to the first fuzzy set of inundated pixels. The resulting fuzzy set has been finally transformed in the flooded area map by marking as inundated every pixel having membership degree greater than 0.5 (the so called defuzzification process to transform a fuzzy number into a crisp number).

## 4. Results

**Figure 8.**
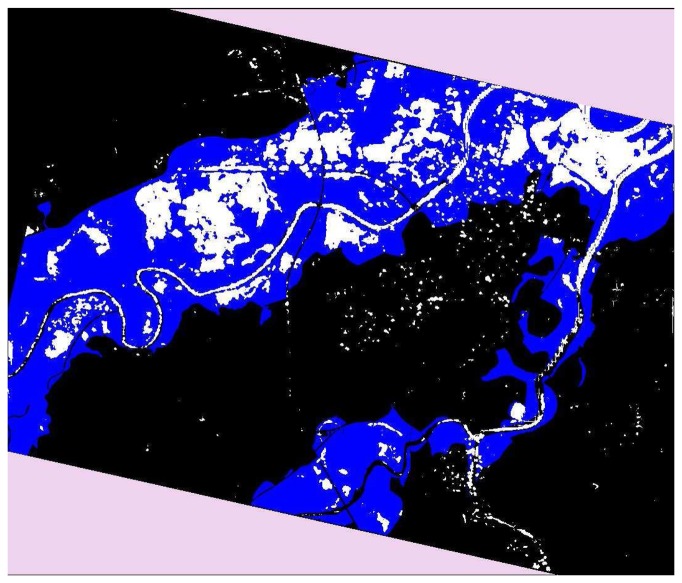
Map of the flooded area (white regions) derived from the fuzzy algorithm. The blue areas represent the inundation according to ground survey (maximum flood extension).

The final result of the procedure is shown in Figure 8. Blue regions correspond to the flooded areas according to the ground survey, whereas white regions represent the flood retrieved by our fuzzy procedure. It is worth underlining that the large blue areas not covered by white ones in Figure 7 do not imply that our algorithm underestimates the inundation. They are due to the temporal mismatch between ground survey and post-flood SAR observation. In other words, because of the late SAR acquisition, only commissioning errors (false alarm) can be quantified.

Among the 771610 pixels of the SAR image, the algorithm has detected about 76000 pixels as flooded, the 87% of which are in agreement with the ground truth classification. The commissioning error is in the order of 5%, whereas the residual 8% corresponds to rivers. The false alarms are represented in Figure 8 by white pixels surrounded by black ones. It is worth noting that in the lower part of the map they are aligned along the Bormida River (affluent of Tanaro), which overflowed as well, so that we suspect that they have been misclassified or skipped during the ground survey. Other commissioning errors are due to pixels which have low radar backscattering in the post-flood SAR image, probably associated to agricultural fields particularly flat or small water basins.

**Figure 9.**
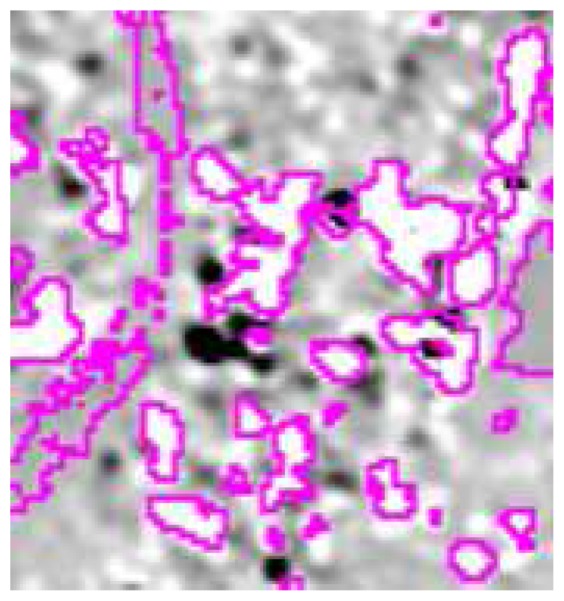
Portion, regarding the city of Alessandria, of the image derived by calculating *σ^0^*(*post*)-*σ^0^*(*pre*). Magenta contours correspond to areas classified as inundated by our algorithm.

It is interesting to analyze the impact of some of the fuzzy rules introduced in the procedure. As discussed in the introduction, a simple thresholding technique, assuming that the water surface acts as a specular reflector, fails where the water surface enhances the double bounce mechanism. This is the case of an urban area. Figure 9 shows a little portion of the image derived by computing the difference between post-flood and pre-flood SAR data. This portion concerns the city of Alessandria. For the sake of figure clarity, the areas classified as inundated by our algorithm are contoured in magenta. Bright pixels correspond to *σ^0^*(*post*) considerably larger than *σ^0^*(*pre*) because of the enhancement of the double bounce backscattering. It can be seen that most of these bright pixels are detected as flooded by the fuzzy classifier. They would have been considered as non-inundated using a thresholding method, or any method not accounting for such a scattering mechanism, while the ground truth agrees with our classification. Note that the grey pixels contoured by magenta lines correspond to the Tanaro river.

**Figure 10.**
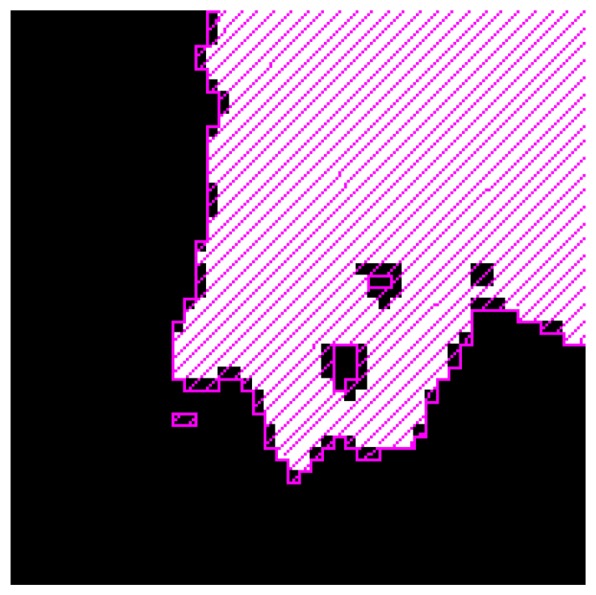
Effect of the inclusion of the DEM information in the classification procedure. Flood maps obtained by either including (polygons filled with magenta lines) or discarding (white pixels) this information are compared.

Being the Alessandria district a plain region, we can expect that the information provided by the DEM has a small impact on the classification result. Nevertheless, we believe that a map of flooded areas should account for hydraulic considerations about the surface orography. In Figure 10, we show a portion of the map, concerning a flooded area according to ground truth, in which the effect of including the DEM information in the classification procedure can be evaluated. The polygons filled with magenta lines represent the zones classified as flooded if the DEM information is taken into account. White pixels correspond to the results achieved by neglecting the DEM. It can be observed that the consideration of the DEM widens the region classified as flooded. Although this widening is small, it produces a better agreement with the ground truth.

## 5. Conclusions

A method based on a fuzzy classification approach has been applied to the problem of flood mapping from SAR imagery. The method accounts for the surface characteristics of the area involved in the inundation, exploiting the information extracted from a Digital Elevation Model. The type of land cover is also taken into account by considering two different scattering mechanisms, i.e., the specular reflection, occurring in open water, and the double bounce effect, occurring in flooded urban and forested areas. Contextual analyses on the neighboring pixels are also introduced to avoid producing noisy maps. The algorithm has been tuned and tested on the inundation occurred in the Alessandria district (Northern Italy) on November 1994. Although the accuracy of the final map cannot be assessed, since the SAR post-inundation acquisition refers to 3 days after the ground survey, while the latter regards the peak of the inundation, the results seems to be encouraging, with the 87% of pixels correctly classified as flooded and a 5% of false alarm. Due to the mentioned unavailability of ground truth at the time of the SAR overpass, it is likely the procedure may require some better tuning of the parameters of the membership functions. However, the proposed scheme based on the fuzzy approach can represent a useful tool to integrate different sources of information and classification rules within an operational system for disaster management.
